# An upper-crust lid over the Long Valley magma chamber

**DOI:** 10.1126/sciadv.adi9878

**Published:** 2023-10-18

**Authors:** Ettore Biondi, Weiqiang Zhu, Jiaxuan Li, Ethan F. Williams, Zhongwen Zhan

**Affiliations:** California Institute of Technology, Seismological Laboratory, Pasadena, CA, USA.

## Abstract

Geophysical characterization of calderas is fundamental in assessing their potential for future catastrophic volcanic eruptions. The mechanism behind the unrest of Long Valley Caldera in California remains highly debated, with recent periods of uplift and seismicity driven either by the release of aqueous fluids from the magma chamber or by the intrusion of magma into the upper crust. We use distributed acoustic sensing data recorded along a 100-kilometer fiber-optic cable traversing the caldera to image its subsurface structure. Our images highlight a definite separation between the shallow hydrothermal system and the large magma chamber located at ~12-kilometer depth. The combination of the geological evidence with our results shows how fluids exsolved through second boiling provide the source of the observed uplift and seismicity.

## Teaser

Fiber-seismic tomography reveals an upper-crust lid over the Long Valley volcanic system.

## INTRODUCTION

Calderas often remain active long after their formation as shown by their surface activity (*1*, *2*), such as fumaroles and large-scale hydrothermal systems. To evaluate the risk of major eruptions, it is critical to characterize the connectivity between surface features and subsurface structures, especially to estimate the volume of potentially eruptible material (*3*, *4*). For example, tomographic images of the Yellowstone Caldera show an upper-crust reservoir of 10,000 km^3^ with an estimated melt fraction varying between 10 and 20% (*5*, *6*) that, under certain circumstances, can produce eruptions one to two orders of magnitude larger than historically observed volcanic events (*7*).

The Long Valley Caldera, located in the Eastern Sierra Nevada mountains of California, is one of the largest calderas in North America and was formed approximately 767 ka ago by a single eruptive event that released 650 km^3^ of rhyolitic material (*8*, *9*). Since 1978, the area has experienced multiple periods of pronounced unrest (*10*). During these periods, the caldera activity included crustal earthquake swarms and sequences (*11*–*13*), long-period volcanic earthquakes (*14*, *15*), surface deformation (mainly inflation) (*16*–*18*), and elevated efflux of magmatic gases (*19*–*22*). Despite substantial efforts to understand the nature of the caldera’s unrest, the mechanism is still debated (*23*). Two competing hypotheses have been proposed: (i) upper-crust magmatic intrusion(s) or (ii) ascending fluids released by second boiling of the rhyolitic reservoir terminally crystallizing at depths greater than 10 km (*24*). Second boiling occurs when a magma body has stopped rising toward the surface and is emplaced in the upper crust at a depth influenced by neutral buoyancy and roof-rock strength. The crystallization of such a body reduces the solubilities of the contained volatiles within the magma mush (e.g., H_2_O and CO_2_) and causes them to be released/exsolved in the form of bubbles or vesicles that rise toward the surface due to buoyancy. On the one hand, relatively low borehole temperatures and lack of observed CO_2_ or He anomalies at the surface indicate a volcanic system whose activity is driven by second boiling (*19*, *20*, *25*). On the other hand, deformations consistent with a dilating spheroidal body at approximately 8-km depth and regular volcanic earthquakes suggest the potential involvement of magma and volatile exsolution from the magma reservoir (*17*, *23*, *25*, *26*). Last, for large rhyolitic systems, such as the Long Valley Caldera, eruptions are typically driven by melt last stored at upper crust depths (for the Long Valley Caldera, ~3 to 8 km) (*27*). Given the complexity of this system, new upper-crust injections and fluid exsolution by second boiling could act in tandem to induce the observed surface deformation and seismicity (*28*). Therefore, the ability to exclude the presence of large-scale upper-crust melt reservoirs would enable a better characterization of the hazard of this volcanic system. To summarize, upper-crust magmatic intrusions would indicate an elevated potential for eruptive activity, while the second boiling nature of the unrest would imply a moribund magmatic system, still hazardous but not as dangerous.

## RESULTS

**Fig. 1.**
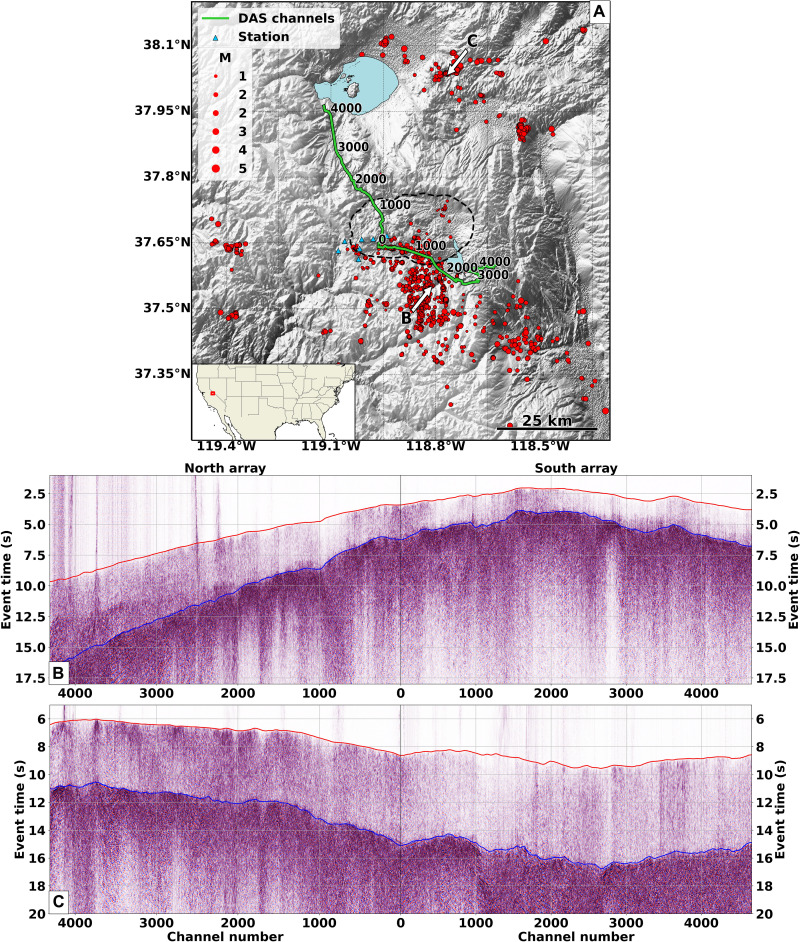
Study area and local and regional events from DAS array. (**A**) Map of the study area in which the distributed acoustic sensing (DAS) channels (green line), seismic stations (blue triangles), and earthquakes (red dots) are indicated. The black dashed line delineates the limit of the Long Valley Caldera. The white arrows point to the two events shown in the bottom panels. The red box in the map inset indicates the study area within the United States. (**B** and **C**) Strain recorded by the DAS arrays induced by local events with Northern California Earthquake Data Center (NCEDC) double-difference (DD) catalog IDs 73482516 and 73491170, respectively. The red and blue curves in these panels show the *P*- and *S*-wave neural network–picked travel times on these two events, respectively. M, Magnitude.

Seismic tomography is a valuable tool to resolve this dichotomy. Recent tomographic studies of the Long Valley Caldera highlighted the presence of a large magmatic body in the middle crust but were limited by the scale and resolution of the seismic arrays used to form the tomographic images (*23*, *29*–*32*). In this study, we use two distributed acoustic sensing (DAS) units to record seismic data along telecommunication fiber-optic cables (Fig. 1 and fig. S1). DAS deployed on fiber-optic cables provides a novel methodology to record earthquakes and other seismic signals with unprecedented temporal and spatial resolution (*33*, *34*), especially in volcanic areas (*35*–*37*). Our two DAS arrays are composed of more than 9000 channels covering an approximately 100-km north-south transect across the caldera, with precise channel locations obtained using a vehicle-based positioning method (*38*). The total aperture and channel density of the DAS arrays enable the imaging of subsurface structures that could not be resolved by previous studies that only relied on conventional networks.

Over a 12-month period, we detected more than 6000 local and regional earthquakes that were also cataloged by the Northern California Earthquake Data Center (NCEDC) (*39*). We trained a deep neural network model to accurately pick more than 12 million *P*- and *S*-wave arrival times and incorporate these measurements within an efficient tomographic workflow. DAS provides a total number of travel-time measurements that is two to three orders of magnitude larger than conventional, even dense, seismic arrays, which represents a computational challenge for existing tomographic approaches. Moreover, volcanic areas present subsurface structures with substantial velocity contrasts resulting in complex seismic raypaths. To properly take into account complex ray geometries and handle the large number of travel-time measurements, we develop a double-difference (DD) Eikonal travel-time tomography workflow based on the adjoint-state method. The nonlinear iterative nature of our method correctly models ray bending, while the matrix-free formulation permits the efficient inversion of billions of DD travel times.

### The Long Valley Caldera shallow structures

**Fig. 2.**
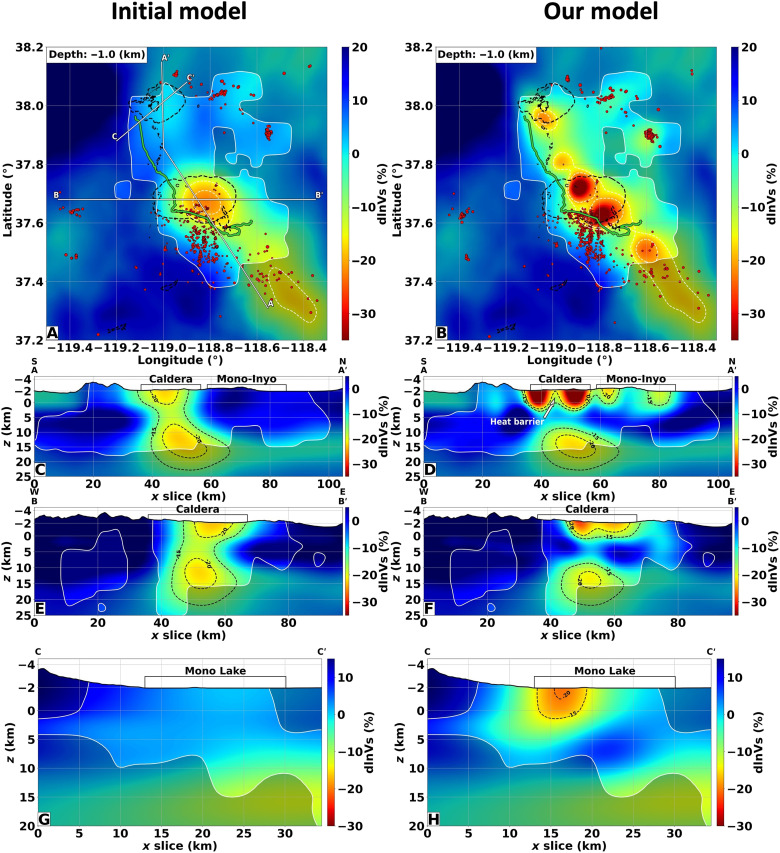
The Long Valley shear-wave anomalies. The panels on the left column display the initial model derived from surface-wave inversion, while the panels on the right depict the inverted *S*-wave velocity anomalies obtained by our tomographic DAS workflow. All perturbations are with respect to a one-dimensional Walker Lane crust profile (obtained by averaging the initial model along latitude and longitude). (**A** and **B**) Depth slices at −1.0 km elevation. The caldera and lakes’ extents are shown by the black dashed lines. (**C** to **H**) Model profiles indicated in (A). The white (A and B) and black (C to H) dashed lines delineate the −20 and −15% *P*-wave velocity contours. The white solid lines separating the shaded areas denote the resolvable model portions.

**Fig. 3.**
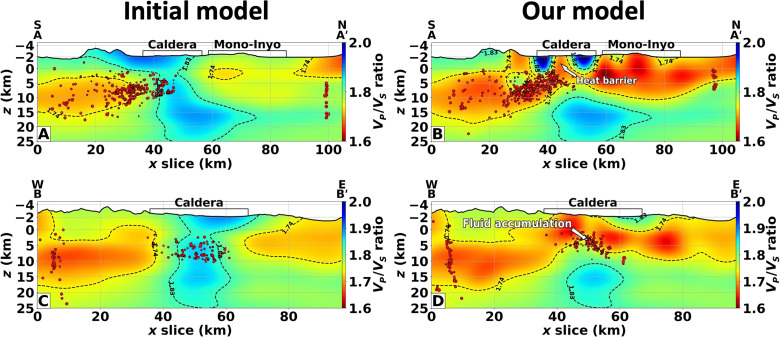
The Long Valley *V_P_*/*V_S_* ratio structures. (**A** and **C**) *V_P_*/*V_S_* ratio derived from the initial wave-speed models for the cross sections AA′ and BB′ in Fig. 1A. (**B** and **D**) Same as the previous panels but obtained from the final models. In all these panels, the red dots indicate the initial and relocated earthquakes within 10 km of the cross sections.

Figure 2 shows our tomographic images of the Long Valley Caldera, in a side-by-side comparison with the latest tomography *P*-wave (*V_P_*) and *S*-wave (*V_S_*) velocity model, which is also our initial model, based on full waveform inversion of surface waves between 6 and 20 s (*40*). All plots are shown as perturbations with respect to an average one-dimensional (1D) Walker Lane crust profile (fig. S5). With the improved data coverage from the two DAS arrays, we substantially improve the model resolution in the top 15 to 20 km. The heterogeneous shallow structures within the caldera, which only appear as a smooth low-velocity anomaly in the initial model, become sharp in our new tomographic model and correlate well with surface geology. These shallow velocity reductions are likely related to the filling material (e.g., volcanic ashes and alluvial) deposited in the depression and the extensive surface hydrothermal system (*20*, *41*). This hypothesis is corroborated by the high *V_P_*/*V_S_* ratio measured (greater than 1.8) within these anomalies, commonly associated with highly fractured and fluid-permeated rocks (Fig. 3) (*42*). The shallow higher velocity and low *V_P_*/*V_S_* ratio barrier separating the two anomalies, visible in the cross section AA′ (see white arrows in Figs. 2D and 3B), has been also observed by other local tomography studies (*30*, *31*) and could be composed of less fractured crystalline rocks compared to the surrounding units. This structure would explain the low temperature measured at the bottom (approximately 2.5-km depth from the surface) of the Long Valley Exploration Well (*43*), which could inhibit the transfer of heat via convective movement of magmatic volatiles by restricting fluid motion.

### The Mono-Inyo Craters structures

With the DAS arrays extending approximately 30 km north of the caldera rim, our tomography closes the gap in knowledge of upper crust structures across the broader Long Valley magmatic system. North of the caldera region lies the Mono-Inyo Craters, a north-south trend of lava domes and volcanic craters. These craters are the youngest geological features in the Long Valley area, ranging in age from 40,000 to only 250 years ago (*44*). Previous studies have highlighted the existence of gravity and resistivity anomalies that have been interpreted as part of the hydrothermal network of this portion of the volcanic field (*45*, *46*). However, seismic surveys were not able to provide evidence of any velocity variations due to the sparsity of station coverage outside the caldera (*32*). Our model is in good agreement with the resistivity and gravity observations, revealing seismic velocity anomalies associated with these upper-crust structures (AA′ cross section, Fig. 2D and fig. S4D). A basin-oriented north-south low-velocity anomaly is evident below the Mono-Inyo Craters, the depth of which reaches approximately 4 km below sea level. Moreover, two reductions in seismic velocity are located within the Mono basin, with one centered below Mono Lake where the most recent volcanic eruptions in the Long Valley region occurred around 250 years ago (*2*) (CC′ cross section, Fig. 2H and fig. S4H). These two anomalies could be again linked with shallow hydrothermal systems given their relatively high *V_P_*/*V_S_* ratio (approximately 1.8). The depth sensitivity of our tomography is not able to resolve the small intruded magmatic bodies that likely formed these volcanic centers (*29*). However, no conduit is evident connecting these structures to a deeper magmatic source, supporting their now hydrothermal nature.

### The Long Valley volcanic upper-crust lid

In the cross-sectional views of Long Valley Caldera, we observe a clear separation between the large magma body at depth and the upper-crust low-velocity structures (Fig. 2 and fig. S4). The initial *V_P_* and *V_S_* models suggested the existence of an approximately 10- to 15-km-wide conduit connecting the deep magma chamber to the shallow crust. This apparent connectivity in previous models is an artifact of the limited depth resolution of surface-wave inversion methods (*47*). In our images, the structure separating the upper- and mid-crust depths is likely the remnant of the roof block that collapsed as part of the caldera-forming 760-ka eruption. The top interface of the magmatic chamber, located at approximately 8-km depth from the mean sea level, is in agreement with previous depth estimates from reflection studies (*48*–*50*). Furthermore, the crustal block above the magmatic chamber presents a typical crustal *V_P_*/*V_S_* value (approximately 1.7), indicating that the magma body at depth is disconnected from the shallower low-velocity structures of the hydrothermal system throughout the caldera (Fig. 3, B and D). The lower boundary of this structure represents the transition between brittle and ductile rock behavior as suggested by the concentration of the seismicity mostly confined in this layer (Fig. 3, B and D), whose lower limit varies from 10- to 15-km depth outside of the caldera to 5-km depth at the center of Long Valley. The west-east section of Fig. 3D reveals the thinning of this layer at about 50 km along the section beneath the resurgent dome at the center of the caldera, with a concentration of seismicity at 4-km depth. The extent and position of this structure correlate well with the geodetic source of recent uplift (*51*). These findings exclude the possibility of shallow intruded magma bodies larger than 2 km and support the interpretation of deformation driven by the accumulation of exsolved fluids at the center of the caldera that permeate the preexisting southern moat and ring faults, driving the observed seismicity. This interpretation is corroborated by the hypothesis-driven tests of figs. S9 to S11 in which any upper-crust velocity reduction larger than 2 km in size can be correctly detected and estimated.

## DISCUSSION

### Key improvements in crustal imaging

The spatial extent and channel density of our DAS arrays overcome the inherent limitations associated with conventional broadband networks often used in body-wave tomography (*4*). Leveraging the DAS high-spatial sampling capabilities, we attain exceptional lateral resolution in shallow depths (0 to 8 km), while the wide aperture of our arrays enables us to image the middle and lower portions of the subsurface (8 to 30 km) with a remarkable level of detail. Our findings exhibit resemblances to earlier studies (*30*, *31*), such as the high-velocity barrier at the center of the resurgent dome (Fig. 2D) and the low velocity and high *V_P_*/*V_S_* ratio shallow anomalies associated with the hydrothermal caldera system (Figs. 2, B and D, and 3B). However, thanks to the aforementioned advantages, we enhance the current understanding by presenting a comprehensive picture of the entire volcanic system, which was missing from previous tomographic results.

**Fig. 4.**
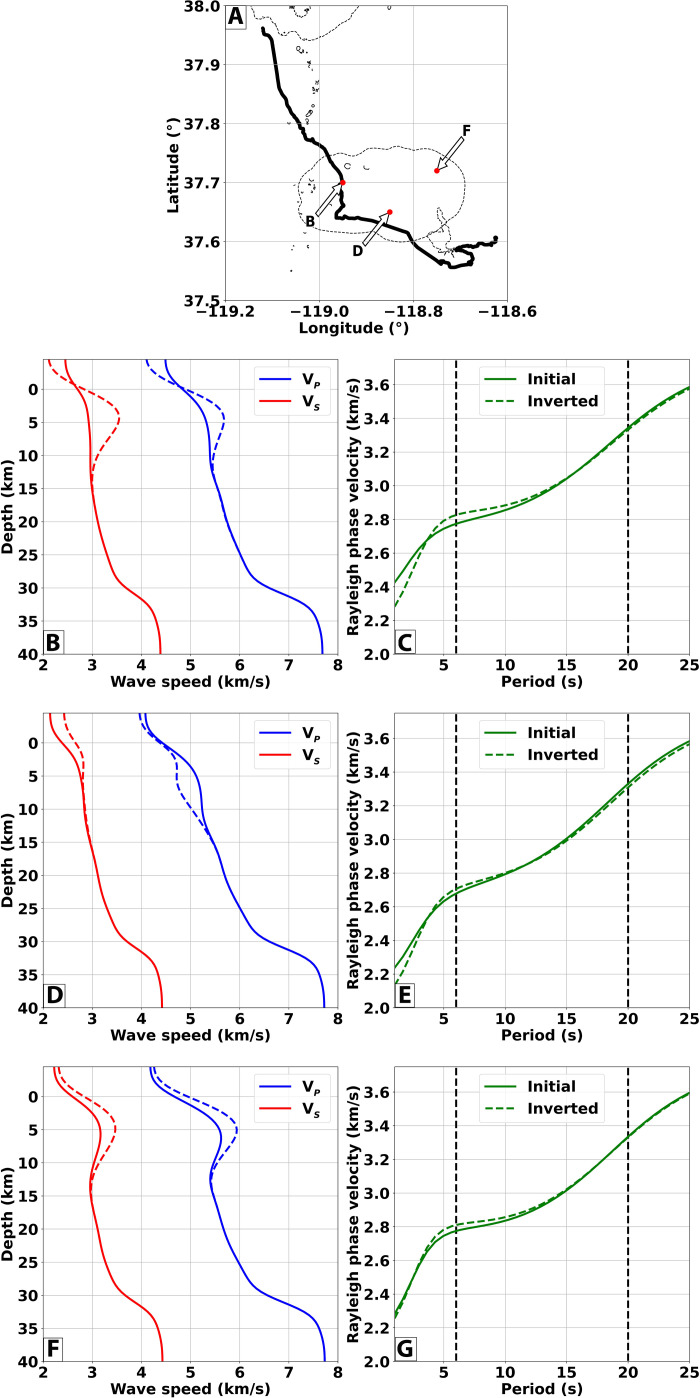
Surface-wave dispersion curves from initial and inverted models. (**A**) Map showing the locations for which the surface-wave dispersion curves are computed. (**B**, **D**, and **F**) *V_P_* (red lines) and *V_S_* profiles of the initial (solid lines) and inverted (dashed line) models extracted at the corresponding points in (A). (**C**, **E**, and **G**) Rayleigh dispersion curves for the corresponding panels on the left evaluated using the initial (solid green line) and inverted (dashed green lines) velocity profiles. The black vertical lines indicate the period range used to construct the initial velocity models [i.e., 6 to 20 s periods; (*40*)].

Similarly, surface-wave inversion strategies can resolve large-scale velocity anomalies but lack the lateral resolution to delineate near-surface structures due to the commonly considered wave periods and station coverage (*43*). This limitation can be verified by comparing surface-wave dispersion curves obtained using the initial and inverted models. To this end, we extract three velocity profiles from locations placed within the caldera area (Fig. 4A) and compute the Rayleigh phase-velocity dispersion curves (*52*). The necessary density profiles are obtained using an empirical relationship calibrated on crustal rocks (*53*). The left panels in Fig. 4 depict the *V_P_* (red lines) and *V_S_* profiles of the initial (solid lines) and inverted (dashed line). The corresponding surface-wave dispersion curves are shown on the right panels in Fig. 4. The black dashed vertical lines bound the range of periods that were used to obtain the initial velocity models. When comparing the dispersion curves from the initial (solid green lines) and the final dashed green lines) models, only minor differences can be observed, and, even at shorter periods than 5 s down to 1 s, the two curves do not present substantial phase velocity differences. This comparison highlights the consistency of the inverted wave speeds in preserving the surface-wave structures and the inability of surface-wave inversion methodologies to refine the near-surface structures due to an intrinsic nonuniqueness of the inverse problem for the considered periods.

### The upper-crust lid confining the exsolved fluids

**Fig. 5.**
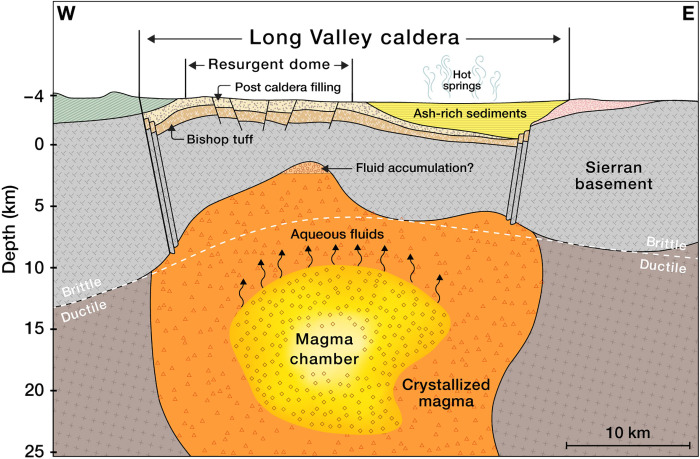
Schematic model of the Long Valley magmatic system interpreted from the tomographic sections. The orientation of this interpretation is along the cross section BB′ in Fig. 3D. The shallow formations are based on the geologic section in (*24*). The location of the ash-rich sediments with a high *V_P_*/*V_S_* ratio and reduced wave speeds correlates well with the hydrothermal activity present in the eastern caldera area (*20*). The boundaries of the Sierran basin follow the 1.74 *V_P_*/*V_S_* ratio bottom contour in Fig. 3D.

Our results reinforce the fluid-driven nature of the uplift and unrest occurring in the Long Valley Caldera and represent the first tomographic evidence supporting the second-boiling hypothesis with a lack of recent upper crust intrusions. Figure 5 shows a schematic model based on the structures highlighted in the BB′ cross section. The resurgent dome presents lower *V_P_*/*V_S_* and higher velocities than the surrounding region, as observed by previous studies (*30*, *31*). The higher *V_P_*/*V_S_* values and lower velocities in the eastern portion of the caldera correlate well with the location of hot springs and ash-rich sediments. In our interpretation, the Sierran basement, which was part of the pre-caldera magmatic roof block, covers the contemporary magma chamber and isolates the magma body from the shallow crust. Our new observations place tighter constraints on the melt region, which exhibits an overall VS anomaly of −15% and a total volume of 6400 km^3^, which is in the same order of magnitude as other large volcanic systems (*5*–*7*). By using experimental melt-fraction curves (*32*), the melt fraction varies from 21 to 23% and corresponds to a total storage of 1350 km^3^ of melt, which agrees with previous estimates (*32*). Within sill-like structures, inferred from the estimated seismic anisotropy in this volcanic system (*54*), the melt fraction might be slightly underestimated compared to the one obtained from the average inverted *V_S_* values. Such melt-fraction values are not close to the critical porosity of a magmatic mush [approximately 40%; (*4*)] required to induce the mobilization of magma toward the surface. Thus, the retrieved velocity anomalies suggest a current textural equilibrium (as a distributed melt or as small melt-rich pockets) of the crystal mush, which implies the stagnation and crystallization of the mid-crust chamber associated with subsequent exsolution of fluids. Fluids released from the apex of the crystallizing chamber are then trapped at the bottom of the Sierran basement providing the pressure source of the observed uplift. Last, the accumulated fluids migrate laterally toward the southern segment of the ring-fault zone and drive the south-moat observed seismicity. This interpretation does not preclude the possibility of new mantle injections that would perturb the textural equilibrium of the magma chamber, which could result in the revitalization of this moribund volcanic system.

## MATERIALS AND METHODS

### Picking *P*- and *S*-wave arrivals on DAS data using machine learning

Figure S1 depicts the local and regional seismicity used within this study. A total of 2173 events (red dots in fig. S1) from the DD catalog of the NCEDC (*39*) have been employed to form our tomographic images. These earthquakes were recorded by both conventional stations (blue triangles in fig. S1) and DAS arrays (green lines in the same figure) and occurred between November 2020 and November 2021. The minimum magnitude considered is 0.1, while the maximum is 4.96. From these events, we select 843 earthquakes with an average signal-to-noise ratio (SNR) equal to or above 40 dB on the DAS data (examples of the selected events are shown in Fig. 1). We estimate the SNR of each event by computing the noise and signal energy using 2- and 0.8-s windows before and after the initially predicted *P*-wave travel time, respectively. We obtain the necessary *P*- and *S*-wave observed travel times by using a neural network model designed to accurately pick DAS data called PhaseNet-DAS (*55*). This model is based on PhaseNet (*56*), which is a modified U-Net architecture (*57*) with 1D convolutional layers for processing 1D time series of seismic waveforms. We extend this model using 2D convolutional layers to fully exploit both spatial and temporal information of 2D DAS data.

PhaseNet-DAS obtains accurate travel-time picks when high-SNR DAS data are fed into the neural network. Figure 1 (B and C) shows two representative events overlaid with the *P*- and *S*-wave picked travel times. Both curves closely follow the *P*- and *S*-wave onsets clearly observable in these two panels. All the other events present a similar behavior in terms of onset travel-time matching quality. We also quantitatively estimate the picking accuracy using a cross-correlation methodology. We cut a 4-s window around the arrivals obtained by PhaseNet-DAS, apply a band-pass filter between 1 and 10 Hz, and calculate the cross-correlation between event pairs. We estimate the differential time by picking the peaks within the cross-correlation profiles for each channel. To further improve the accuracy of the differential travel-time measurements, we use a multichannel cross-correlation strategy (*58*, *59*) to extract the peaks across multiple cross-correlation profiles. For our analysis, we choose event pairs whose average cross-correlation coefficients are higher than 0.4 for *P* wave and 0.6 for *S* wave. This choice allows us to retrieve 34,193,571 *P*-wave and 3,944,318 *S*-wave differential travel-time measurements from 7583 (fig. S2A) and 1095 event pairs, respectively. The histograms of these differential travel-time values are shown in fig. S2 (B and C). If we assume Gaussian-distributed travel-time picking errors, then the differential time measurements obtained by waveform cross-correlation can be used to estimate the error distribution. The differential travel-time measurements have a mean of −1 ms and an SD of 70 ms for *P* waves and a mean of 3 ms and an SD of 140 ms for *S* waves. These values correspond to SDs of 49.5 and 99 ms for *P*- and *S*-wave picking errors made by the PhaseNet-DAS model, respectively. For comparison, the absolute arrival-time errors of the PhaseNet architecture compared with manual picks on conventional stations have a mean of 2.1 ms and an SD of 51.5 ms for *P* waves and a mean of 3.3 ms and an SD of 82.9 ms for *S* waves (*56*).

### DD Eikonal relocation and tomography

We develop a DD Eikonal travel-time relocation and tomography workflow based on well-established inversion packages (*60*). Compared to these methodologies, our inversion scheme is matrix-free and based on nonlinear optimization iterative schemes. We compute the necessary objective function gradients using the adjoint-state method applied to the Eikonal equation (*61*–*64*). By not storing and inverting any matrix during the optimization process, we are able to invert travel-time picks obtained on the thousands of channels composing the DAS arrays used in this study. Within a DD framework, the size of the considered least-squares matrices would be on the order of billions of elements, which even with modern computational architectures would be impossible to invert within a reasonable time. Because of this limitation, we implement our workflow using an operator-based optimization approach (*65*). To mitigate the location and velocity structure trade-off, we perform the travel-time inversion in an alternate-direction fashion (*63*): We first relocate the events for fixed *P*- and *S*-wave velocity structures, then fix the earthquake locations, and invert the *P*- and *S*-wave models. These two steps are alternated until convergence is reached on the basis of locations and velocity model changes. For the relocation step, we minimize the following objective function

$$\phi (x_{s})=\frac{1}{2}{\left\|\left[\begin{array}{c} \lambda_{A}I\\ \lambda_{\mathrm{DD}}D \end{array}\right][f_{v}(x_{s})+\tau_{0}-\tau_{\mathrm{obs}}]\right\|}_{2}^{2}+\frac{\epsilon }{2}{\left\|x_{s}-x_{s,0}\right\|}_{2}^{2}$$ (1)

where λ_A_ and λ_DD_ are the relative weights of the absolute and DD travel-time errors (*59*), respectively; **τ**_obs_ are the observed *P*- and *S*-wave travel times; ***f_v_*** is the Eikonal operator for fixed velocity structures ***v*** in which reciprocity is used (*63*); ***x**_s_* represents the earthquake locations and τ_0_ represents their origin times; and **D** is the DD operator (*66*), which represents a matrix of size *M* × *N* (*M*, number of DD observations; *N*, number of events). This operator contains the following weighting term

$$W_{ij}=\max^{a}\left[0,1-{\left(\frac{s_{ij}}{c}\right)}^{b}\right]$$ (2)

where *s_ij_* is the interevent distance between the *i*th and *j*th events, *c* is a cutoff value to dismiss measurements of event pairs with an interevent distance larger than its value, and *a* and *b* are exponents that define the shape of the weighting curve. In our workflow, we set *a*, *b*, and *c* to 1.0, 1.0, and 5.0 km, respectively. The regularization term based on the initial earthquake locations ***x***_*s*,0_ and weight by the scalar ε is necessary to avoid inversion instabilities when λ_A_ = 0 (*66*). This regularization term is necessary at the last relocation step when the velocity structures and current event locations are assumed to be close to their correct values. In other cases, we set ε = 0. For each event, the optimal origin time is found at the beginning and at the end of the relocation process with the following equation

$$\tau_{0}^{j}=\frac{1}{N_{r}}\sum_{k=1}^{N_{r}}[\tau_{\mathrm{obs}}^{j,k}-f_{v}^{j,k}(x_{s})]$$ (3)

where optimal origin time for the *j*th event is simply the average of the travel-time residuals over the picked *N_r_* stations.

The velocity models are obtained by minimizing the following cost function

$$\psi (v)=\frac{1}{2}{\left\|\left[\begin{array}{c} \lambda_{A}I\\ \lambda_{\mathrm{DD}}D \end{array}\right][f_{x_{s}}(v)+\tau_{0}-\tau_{\mathrm{obs}}]\right\|}_{2}^{2}$$ (4)

where ***f_x_**__s__* is the Eikonal operator for fixed event locations ***x**_s_*. The operators ***f*** solve the following Eikonal equation

$$\nabla \cdot \nabla \tau =\frac{1}{v^{2}}\;\text{subject to}\;\tau (x_{◼})=0$$ (5)

where ***x***_∎_ represents the event or receiver locations for ***f******_x_****__s__* and ***f*_v_****, respectively. Smoothness in the model is imposed by applying a Gaussian filter to the computed gradient during the optimization process. We use the Broyden–Fletcher–Goldfarb–Shanno (BFGS) (*67*) iterative scheme to minimize both objective functions, and the iteration process is stopped when the considered model parameters are not changing substantially (i.e., less than 1% change compared to the previous iteration).

In this study, event relocation is achieved using *P*- and *S*-wave travel times obtained using PhaseNet (*56*) applied to 80 three-component stations for the 2173 events shown in fig. S1, while the tomography step is conducted using the travel times from the 843 picked by PhaseNet-DAS. We perform the tomography step for *P*- and *S*-wave models independently; thus, the obtained structures in each wave-speed model are inferred by independent data information. The Gaussian smoothing width is based on the results of checkerboard tests conducted at different scales, and we set its SD to 5 km. At the beginning of the inversion process, a higher weight to the absolute travel-time residuals is imposed, and, as the workflow progresses, the relative DD residuals are weighted with higher values (*60*).

## Supplementary Materials

### This PDF file includes:

Supplementary Text Figs. S1 to S11 References
