# Supplementary material for: An upper-crust lid over the Long Valley magma chamber
Source: Sci Adv. 2023 Oct 18;9(42):eadi9878. doi: 10.1126/sciadv.adi9878 (PMC10584340; doi:10.1126/sciadv.adi9878)
Supplement: Supplementary file 1 — Supplementary Text Figs. S1 to S11 References [file sciadv.adi9878_sm.pdf]

Science Advances
AAAS

# Supplementary Materials for

## An upper-crust lid over the Long Valley magma chamber

Ettore Biondi *et al.*

Corresponding author: Ettore Biondi, ebiondi@caltech.edu

*Sci. Adv.* **9**, eadi9878 (2023)
DOI: 10.1126/sciadv.adi9878

**This PDF file includes:**

Supplementary Text
Figs. S1 to S11
References

## Supplementary Text

Checkerboard test and model resolvability

We conduct conventional seismic tomography checkerboard tests in which oscillatory anomalies of -5% and 5% variations in the P- ($V_P$) and S-wave ($V_S$) speeds are introduced within the initial models (40,68). Based on the estimated picking errors, we introduce zero-mean Gaussian noise to the model traveltimes whose standard deviations are 50 ms and 100 ms to the P- and S-wave picks, respectively. We also randomly perturb the event locations by ±500 m in each direction. The panels in Fig. S3 show the true and retrieved perturbations at various depth levels for the inverted $V_S$. To indicate the portions of the models that are resolvable by the tomography approach, we employ the resolvability index (5, 69, 70). This index ranges from 0 to 1: 0.5 represents portions of the model for which 0% of the perturbation is retrieved (i.e., zero sensitivity), a value of 1 is a perfect reconstruction, and 0 defines portions for which -100% of the perturbation is inverted. The shaded areas in these panels indicate a resolvability index smaller than 0.6, which is considered a reasonable lower bound for resolvable areas (5, 70). Similar results are obtained for the $V_P$ perturbations. The best resolution (i.e., no smearing effect) is obtained in the proximity of the DAS channels for most of the depth slices. For the shallow slices (-2 to 2 depths), almost no smearing is observed in the proximity to the sensed fibers. Deeper slices present a broader area of resolvability but at the cost of smearing the perturbations. Below 15 km depth, a significantly reduced portion of the subsurface is resolvable due to the limited number of rays reaching that portion of the model.

P-wave velocity anomalies, residual histograms, and velocity-model validation

Fig. S4 displays the same slices of Fig. 2 in the main text but for the $V_P$ anomalies from a one-dimensional Walker Lane crust velocity model shown in Fig. S5. This one-dimensional model is obtained by averaging the initial model along the latitude and longitude axes. Compared to the initial model shown in the panels on the left column, the inverted velocity anomalies (right column) present the same clear separation between the magmatic chamber centered at approximately 12.5 km depth and the shallow structures above as in the S-wave anomalies. Additionally, similarly to the $V_S$ model, velocity reductions within the caldera, along the Mono-Inyo craters, and underneath the Mono lake are obtained by the tomographic workflow. Similar features are obtained from the tomographic workflow when using this 1D model as an initial guess. However, these anomalies are better defined by starting the tomographic process from the surface-wave inverted velocities. In addition, the 3D surface-wave-derived model as an initial guess provides lower final traveltime residuals compared to the 1D model when used to start the tomography workflow.

The top panels in Fig. S6 show the P- and S-wave absolute traveltime residual histograms obtained using the initial velocity models (Fig. S6 A and B, respectively). Their respective residual means are -0.17 s and 0.11 s, while their standard deviations are 0.65 s and 0.78 s. The relocation and tomography workflow produces velocity models whose traveltime residuals are Gaussian distributed with means of -0.01 and 0.01 and standard deviations of 0.4 s and 0.47 s for P- and S-wave traveltimes, respectively (Fig. S6 C and D). Tighter distributions could be achieved by relaxing the smoothness constraints defined by the Gaussian filter employed during the inversion process.

However, smaller-scale velocity anomalies are not resolvable by the event-channel geometry (based on checkerboard test analyses) and thus we avoid introducing them during the inversion process.

Finally, we validate our inverted velocities by modeling the arrival times for a relocated event that was not included during the tomographic steps. The event ID from the NCEDC DD catalog is 73485976 and its magnitude and relocated depth are 2.8 and 2.327 km, respectively. The maximum and minimum distances from the DAS channels are 33.5 km and 54.5 km (Fig. S7). Fig. S7 displays the recorded DAS data in which we overlay the P- (red lines) and S-wave (blue lines) traveltimes predicted from the initial (dashed lines) and inverted (solid lines) velocity models. The final velocities predict traveltimes closely following the observed arrival onsets. On the other hand, the initial models underestimate the observed onsets, highlighting the presence of low-velocity anomalies (e.g., Mono-Inyo crater basin) necessary to obtain correct traveltime predictions.

Our melt fraction estimations are based on a linearized $V_S$/melt-fraction derivative ($\delta V_S/\delta MF$) of -23 m/s/MF derived by averaging the Voigt and Reuss $V_S$/melt-fraction trends for a 4% H2O- wet rhyolite at 310 MPa and 750 C$^o$ (27). In our models, the -15% and -20% S-wave reductions within the magma chamber correspond to wave speeds of 3.0 km/s and 2.86 km/s with $V_P/V_S$ of 1.83 and 1.86.

Hypothesis-driven resolution tests

To test the resolution and bias of our workflow in imaging known seismic anomalies, we invert synthetic traveltime datasets where different type of velocity reductions. As in other tomographic studies (5, 32), when displaying the results of our synthetic tests, we show the input and inverted anomalies with the background model removed to better highlight the resolved portion of the introduced perturbation.

In our first resolvability test, we introduce a low $V_P$ and $V_S$ anomaly underneath Mono Lake with a high $V_P/V_S$ to simulate the presence of a large water saturated materials (Fig. S8A-D). We apply the same procedure as described for the checkerboard test to invert the synthetic traveltimes. Our tomographic workflow can correctly retrieve the shape and the overall velocity decrease as well as $V_P/V_S$ ratio with a minor underestimation of both properties (Figs. S7E-H).

In our second set of simulated experiments (Figs. S8-S10), we incorporate a sequence of low-shear-wave velocity reductions that progressively diminish in size. The goal is to evaluate the resolution limits of our method in imaging upper-crust magma reservoirs, which are not present within our results obtained using our DAS dataset. In each test we introduce a cylinder-shaped anomaly, a large perturbation of 10 km radius and 4 km thickness (Fig. S9), a middle-size reservoir of 5 km radius and 4 km thickness (Fig. S10), and a small 2 km radius and 2 km thickness anomaly (Fig. S11). In all three cases the shape of the anomalies is well-imaged by our tomographic approach with an underestimation of the velocity decrease within the center of the anomaly; especially, for the smallest anomaly of 2 km radius. If the large and middle-size reservoirs were present within our results, we would have clearly detected and interpreted them as potential upper-crust magma reservoirs. On the contrary, the smallest anomaly is close to the resolution limits of the method given the employed DAS geometry and earthquake locations. Thus, any small upper-crust reservoirs whose core has a size less than 2 km in diameter may be challenging to detect due to the underestimation of the velocity reduction.

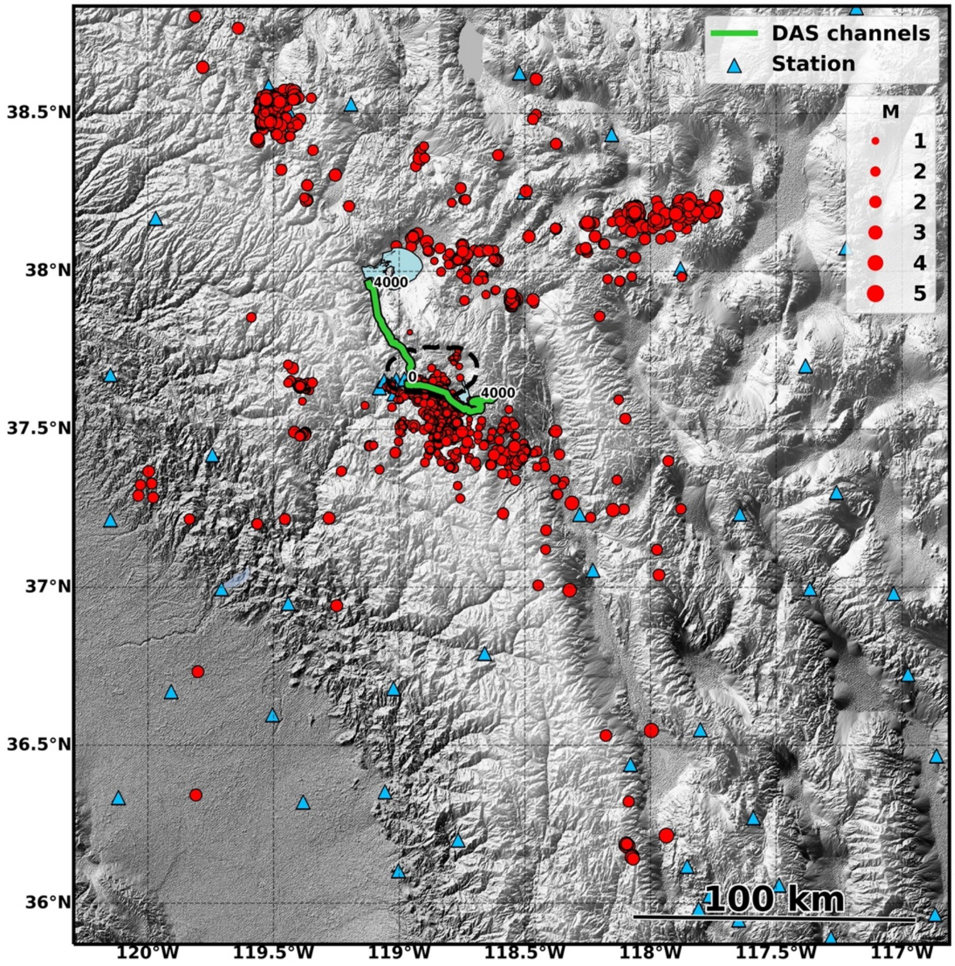


**Fig. S1. Local and regional events used in this study.** Conventional stations are depicted by the blue triangles while the green line indicates the locations of the DAS channels. The earthquakes are indicated by the red dots whose sizes are proportional to their magnitude.

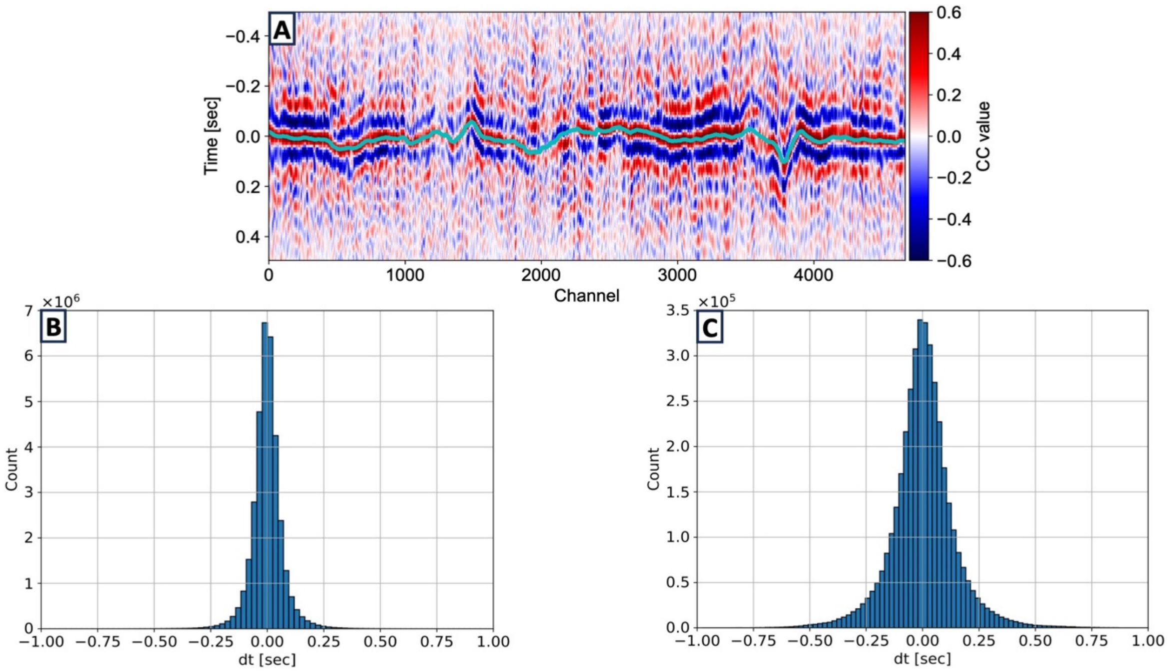


**Fig. S2. Assessing picking accuracy using event cross-correlations.** (**A**) Cross-correlation example between two events recorded by the South DAS array. The cyan line represents the cross-correlation-based shift retrieved by the multi-channel cross-correlation algorithm. (**B** and **C**) Differential traveltime histograms for the P- and S-wave cross-correlation windows, respectively.

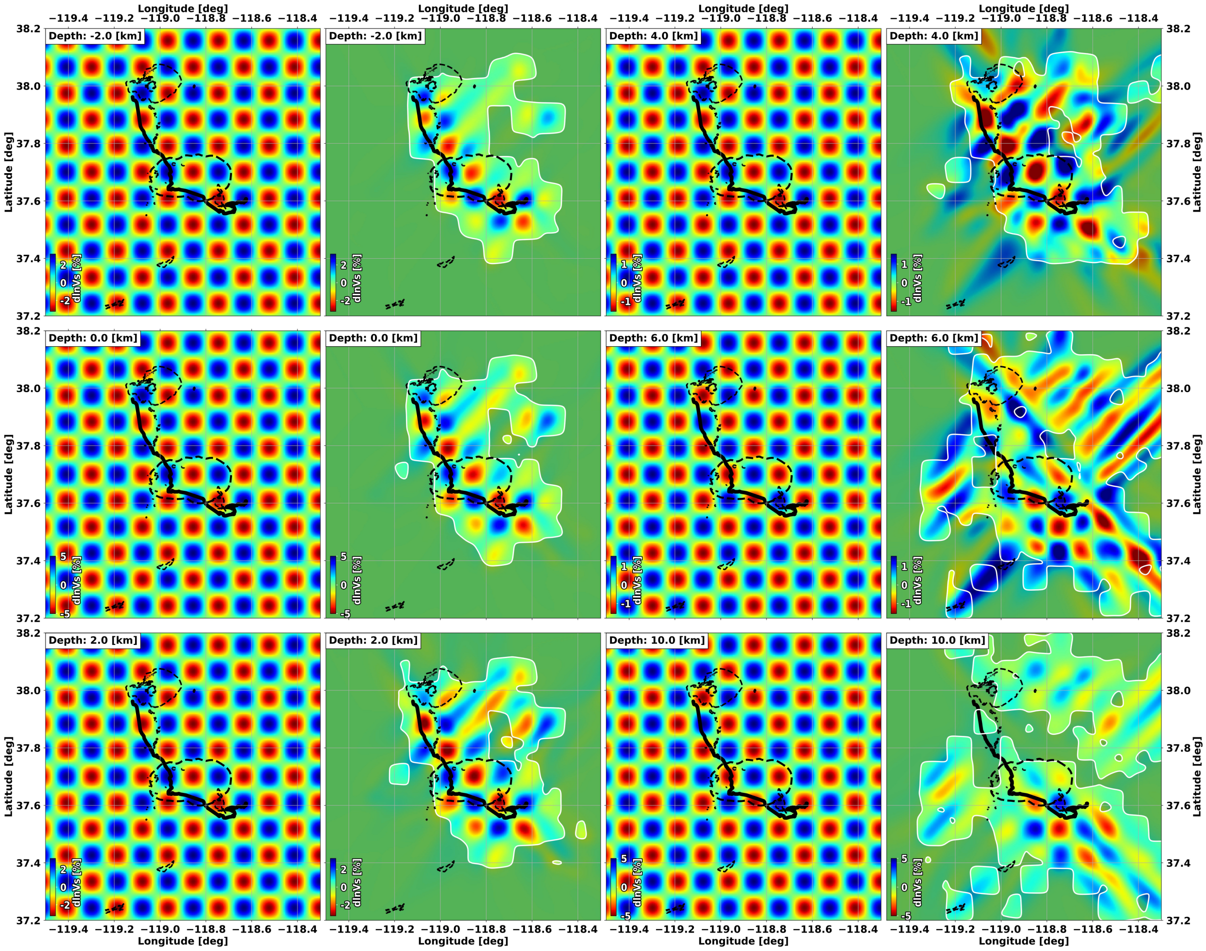


**Fig. S3. Checkerboard test results for the $V_S$ model.** The size of each Gaussian anomaly is 10 km in each direction.

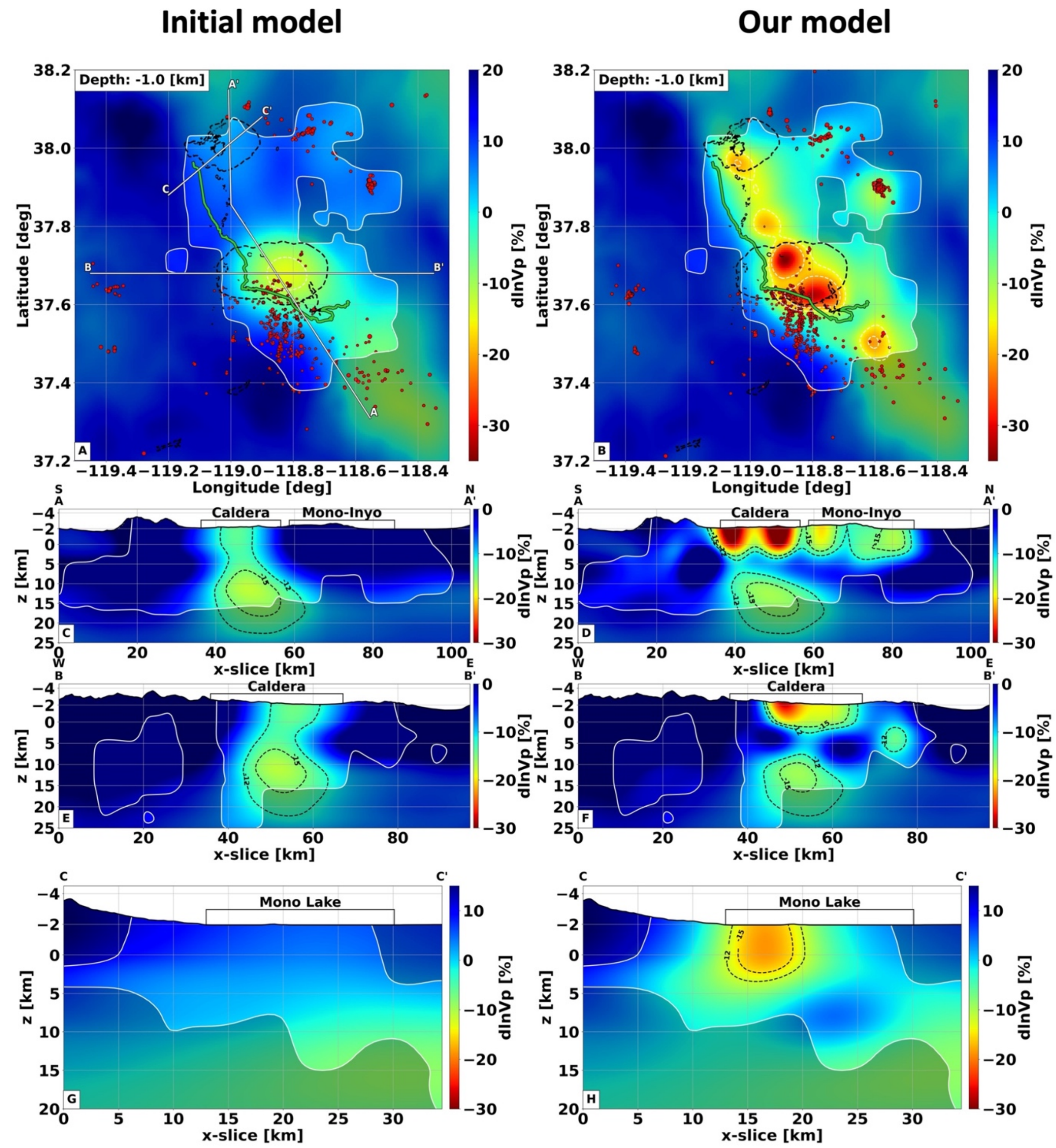


**Fig. S4. The Long Valley P-wave anomalies.** The panels on the left column display the initial model structures, while the panels on the right depict the inverted P-wave velocity anomalies. All perturbations are with respect to a one-dimensional Walker Lane crust profile (obtained by averaging the initial model along latitude and longitude). (**A** to **B**) Depth slices at -1.0 km elevation. The caldera and lakes' extents are shown by the black dashed lines. (**C** to **H**) Model profiles indicated in panel **A**. The white (panels **A** and **B**) and black (panels **C** to **H**) dashed lines indicate the -15% and -12% P-wave velocity contours. The white solid lines separating the shaded areas denote the resolvable model portions.

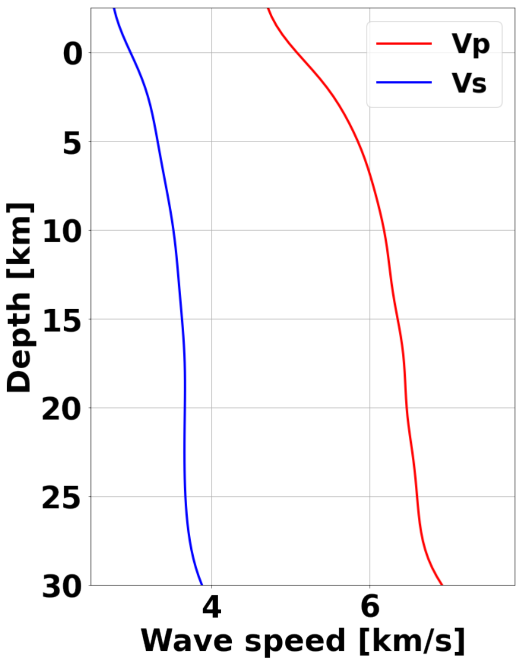


**Fig. S5. Reference Walker Lane velocity profiles.** P- and S-wave speed profiles employed to compute all the velocity perturbations shown in Figs. 2 and S4.

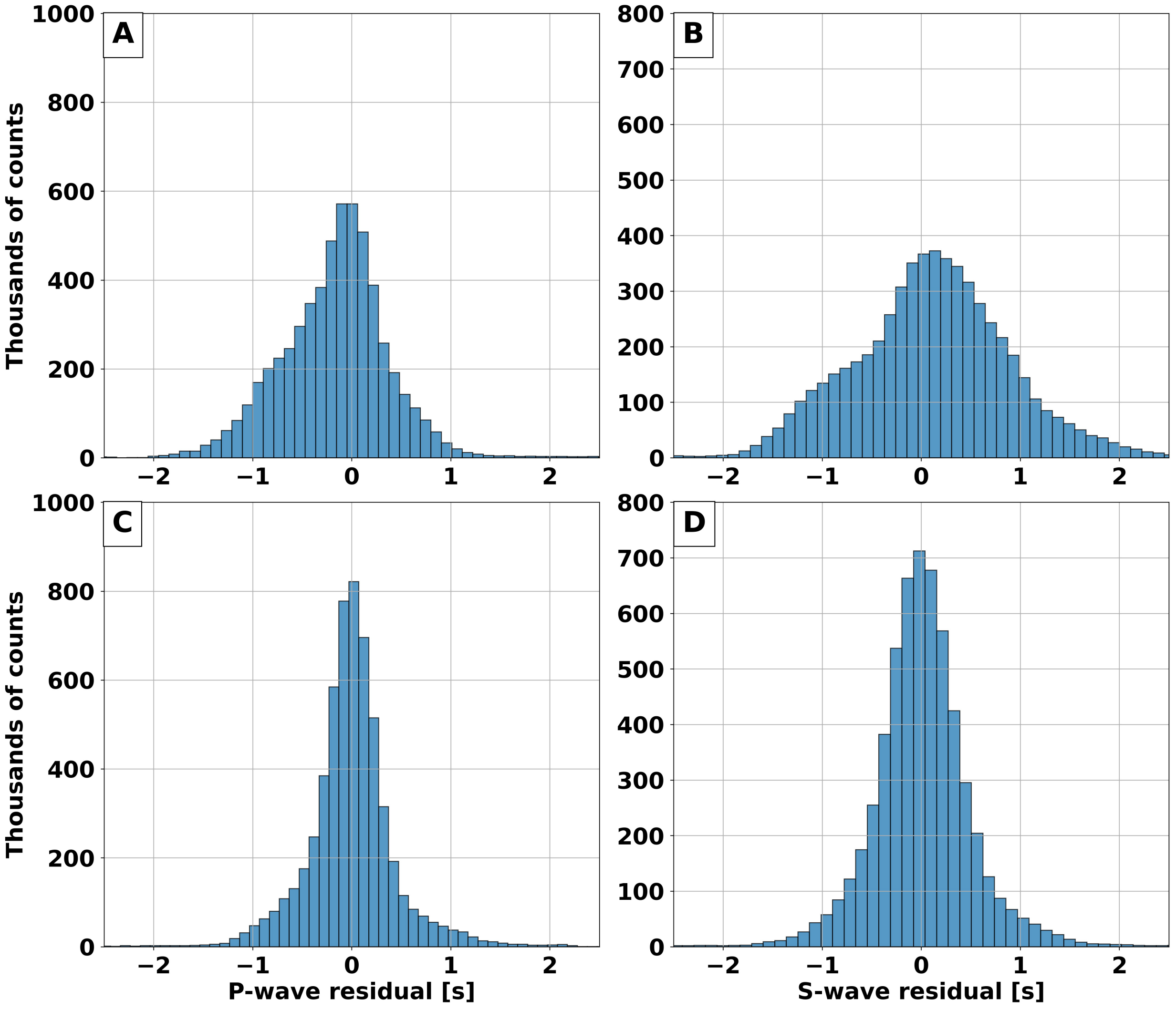


**Fig. S6. Initial and final traveltime residual histograms.** (**A** and **B**) Absolute traveltime residual histograms obtained using the initial P- and S-wave speed models. (**C** and **D**) Same as the top panels but obtained with traveltimes predicted using the inverted models.

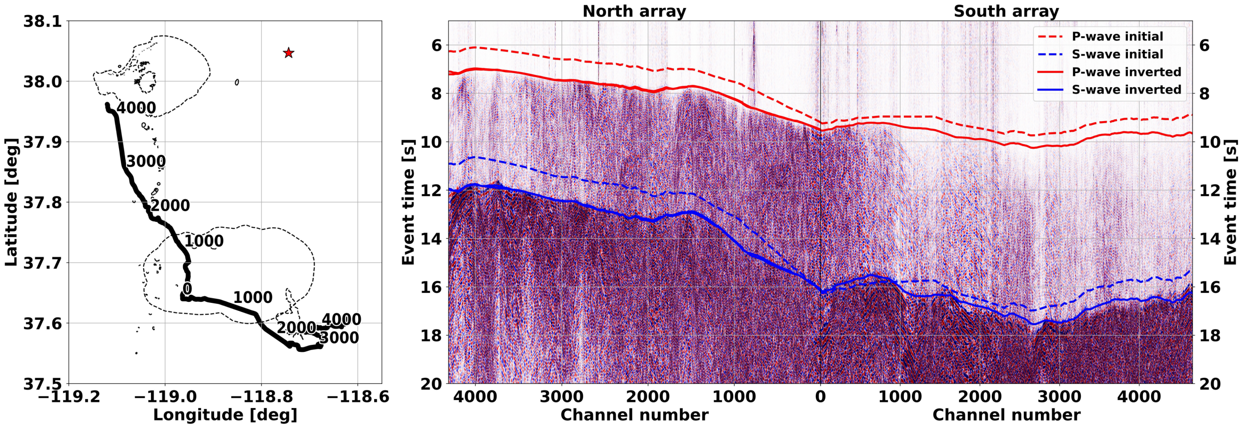


**Fig. S7. Inverted wave-speed assessment with an independent event.** (Left) Map showing the location of the DAS array channels (black solid line) and of the event (red star) used for result validation. Recorded DAS strain of the event overlaid by the P- (red lines) and S-wave (blue lines) predicted arrival times using the initial (dashed lines) and inverted (solid lines) models (Right).

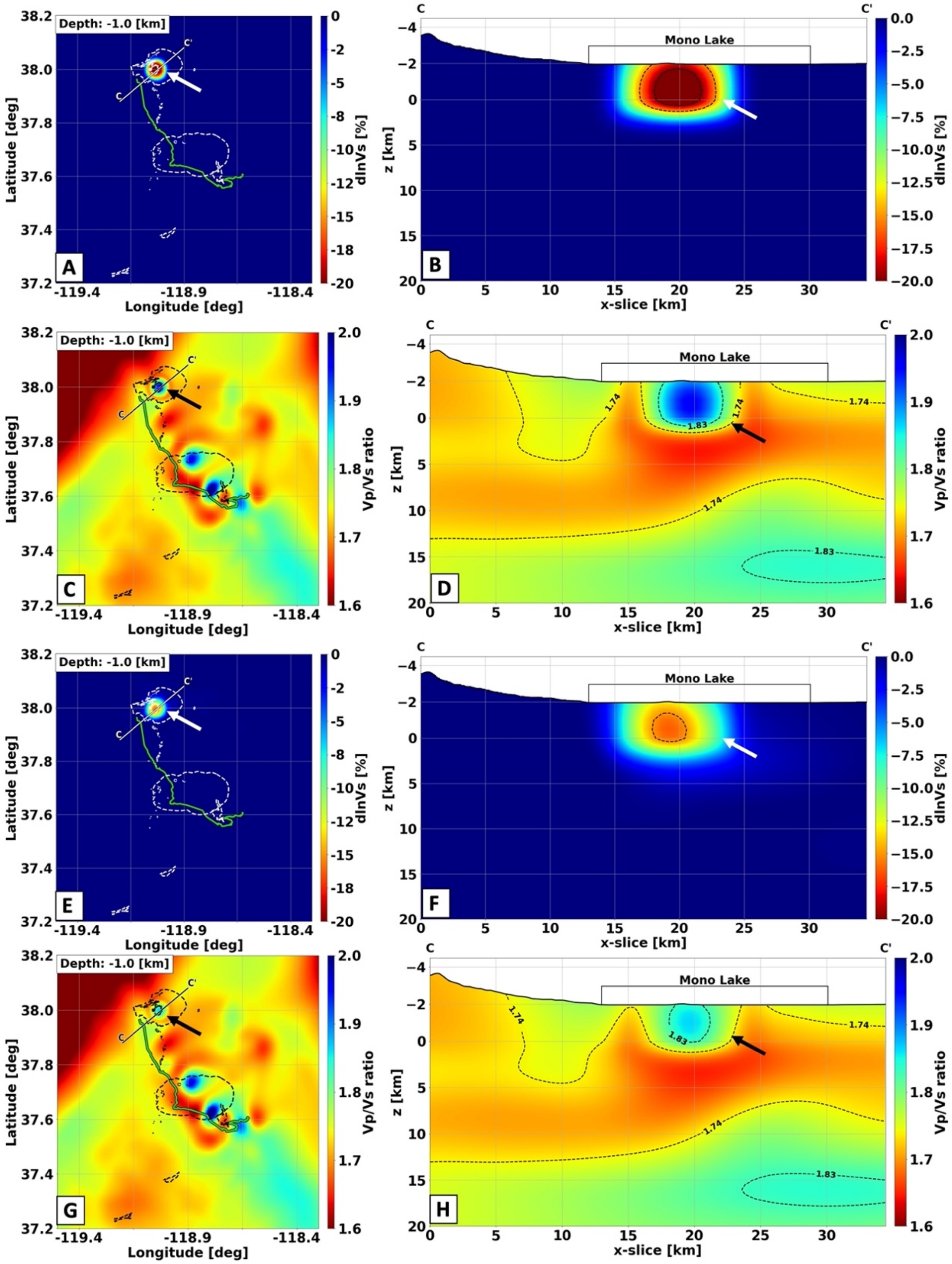


**Fig. S8. Resolvability test for low velocities and high $V_P/V_S$ anomaly under Mono Lake.** (**A-D**) $V_S$ decrease and $V_P/V_S$ ratio anomaly introduced within our model to test the resolvability of the tomographic workflow underneath Mono Lake. A similar synthetic $V_P$ reduction is placed under Mono Lake. (**E-H**) $V_S$ reduction and $V_P/V_S$ ratio anomaly retrieved by our method. The arrows point at the introduced and recovered anomaly in each panel. The black dashed lines in panels **B** and **F** indicate -15% and -20% velocity reductions.

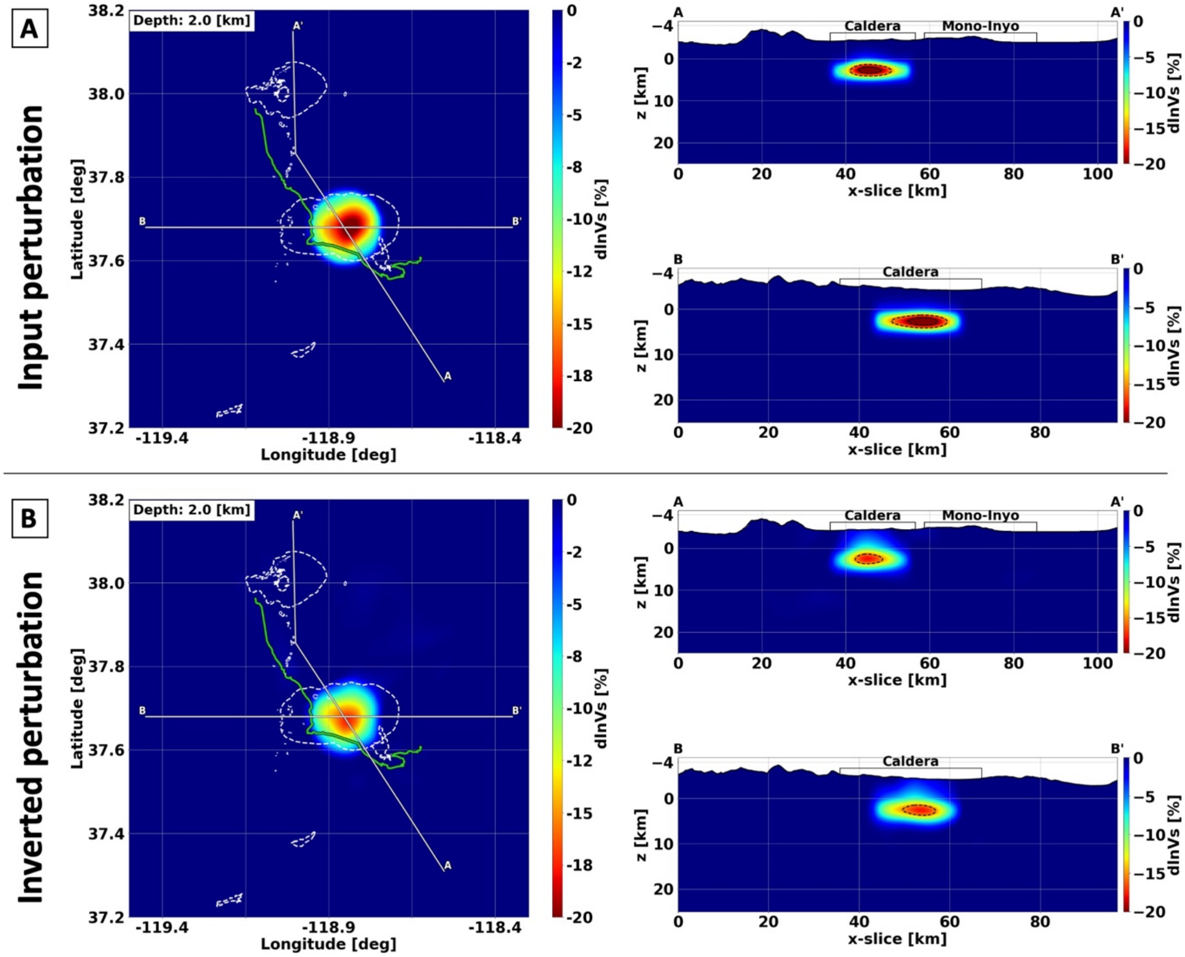


**Fig. S9. Resolvability test for an upper-crust low-velocity anomaly under the Long Valley caldera of 10 km radius.** (**A**) Input synthetic $V_S$ anomaly mimicking an upper-crust magma reservoir. (**B**) Velocity anomaly retrieved by our tomographic workflow. The black dashed lines in the velocity profiles indicate -15% and -20% velocity reductions.

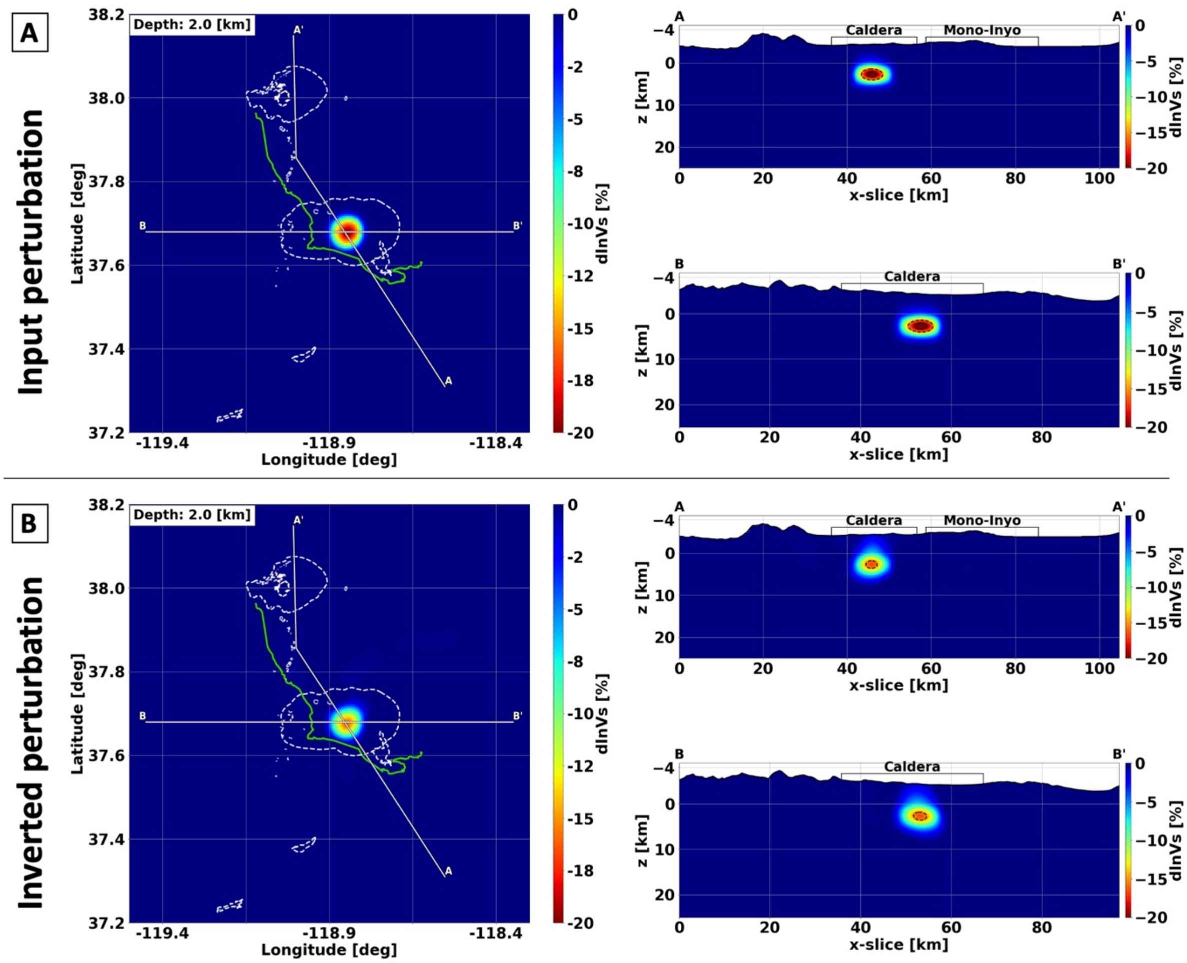


**Fig. S10. Resolvability test for an upper-crust low-velocity anomaly under the Long Valley caldera of 5 km radius.** (**A**) Input synthetic $V_S$ anomaly mimicking an upper-crust magma reservoir. (**B**) Velocity anomaly retrieved by our tomographic workflow. The black dashed lines in the velocity profiles indicate -15% and -20% velocity reductions.

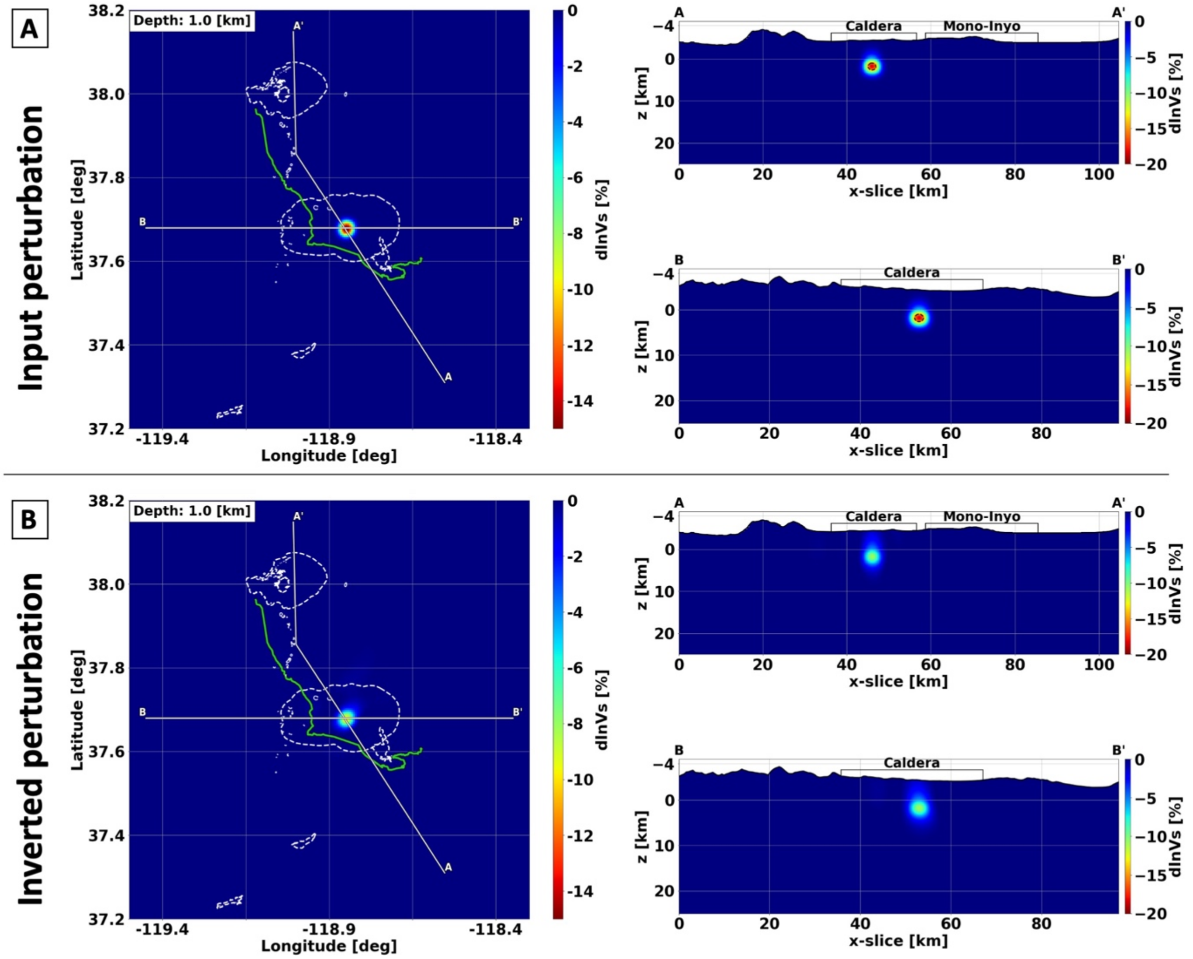


**Fig. S11. Resolvability test for an upper-crust low-velocity anomaly under the Long Valley caldera of 2 km radius.** (**A**) Input synthetic $V_S$ anomaly mimicking an upper-crust magma reservoir. (**B**) Velocity anomaly retrieved by our tomographic workflow. The black dashed line in the velocity profiles indicates a -15% velocity reduction.

## REFERENCES AND NOTES


1. J. B. Lowenstern, R. B. Smith, D. P. Hill, Monitoring super-volcanoes: Geophysical and geochemical signals at Yellowstone and other large caldera system. *Philos. Trans. Math. Phys. Eng. Sci.* **364**, 2055–2072 (2006).

2. A. Bevilacqua, M. Bursik, A. Patra, E. B. Pitman, Q. Yang, R. Sangani, S. Kobs-Nawotniak, Late quaternary eruption record and probability of future volcanic eruptions in the Long Valley Volcanic region (CA, USA). *J. Geophys. Res. Solid Earth* **123**, 5466–5494 (2018).

3. National Academies of Sciences, Engineering, and Medicine, *Volcanic Eruptions and Their Repose, Unrest, Precursors, and Timing* (National Academies Press, 2017).

4. M. Paulatto, et al., *EarthArXiv* (2022).

5. H.-H. Huang, F.-C. Lin, B. Schmandt, J. Farrell, R. B. Smith, V. C. Tsai, The Yellowstone magmatic system from the mantle plume to the upper crust. *Science* **348**, 773–776 (2015).

6. R. Maguire, B. Schmandt, J. Li, C. Jiang, G. Li, J. Wilgus, M. Chen, Magma accumulation at depths of prior rhyolite storage beneath Yellowstone Caldera. *Science* **378**, 1001–1004 (2022).

7. C. J. N. Wilson, G. F. Cooper, K. J. Chamberlain, S. J. Barker, M. L. Myers, F. Illsley-Kemp, J. Farrell, No single model for supersized eruptions and their magma bodies. *Nat. Rev. Earth Environ.* **2**, 610–627 (2021).

8. R. A. Bailey, G. B. Dalrymple, M. A. Lanphere, Volcanism, structure, and geochronology of Long Valley Caldera, Mono County, California. *J. Geophys. Res.* **81**, 725–744 (1976).

9. W. Hildreth, C. J. Wilson, Compositional zoning of the bishop tuff. *J. Petrol.* **48**, 951–999 (2007).

10. D. P. Hill, Unrest in Long Valley Caldera, California, 1978–2004. *Geol. Soc. Spec. Publ.* **269**, 1–24 (2006).

11. J. C. Savage, R. S. Cockerham, Earthquake swarm in Long Valley Caldera, California, January 1983: Evidence for dike inflation. *J. Geophys. Res. Solid Earth* **89**, 8315 (1984).

12. E. Montgomery-Brown, D. R. Shelly, P. A. Hsieh, Snowmelt-triggered earthquake swarms at the margin of Long Valley Caldera, California. *Geophys. Res. Lett.* **46**, 3698–3705 (2019).

13. B. Q. Li, J. D. Smith, Z. E. Ross, Basal nucleation and the prevalence of ascending swarms in Long Valley caldera. *Sci. Adv.* **7**, eabi8368 (2021).

14. A. Pitt, D. Hill, Long-period earthquakes in the Long Valley Caldera Region, eastern California. *Geophys. Res. Lett.* **21**, 1679–1682 (1994).

15. E. E. Brodsky, S. G. Prejean, New constraints on mechanisms of remotely triggered seismicity at Long Valley Caldera. *J. Geophys. Res. Solid Earth* **110**, B04302 (2005).

16. D. S. Dreger, H. Tkalcic, M. Johnston, Dilational processes accompanying earthquakes in the Long Valley Caldera. *Science* **288**, 122–125 (2000).

17. M. Battaglia, P. Segall, C. Roberts, The mechanics of unrest at Long Valley caldera, California. 2. Constraining the nature of the source using geodetic and micro-gravity data. *J. Volcanol. Geotherm. Res.* **127**, 219–245 (2003).

18. P. Tizzani, M. Battaglia, G. Zeni, S. Atzori, P. Berardino, R. Lanari, Uplift and magma intrusion at Long Valley caldera from InSAR and gravity measurements. *Geology* **37**, 63–66 (2009).

19. M. Sorey, B. Kennedy, W. Evans, C. Farrar, G. Suemnicht, Helium isotope and gas discharge variations associated with crustal unrest in Long Valley Caldera, California, 1989–1992. *J. Geophys. Res. Solid Earth* **98**, 15871–15889 (1993).

20. M. L. Sorey, G. A. Suemnicht, N. C. Sturchio, G. A. Nordquist, New evidence on the hydrothermal system in Long Valley caldera, California, from wells, fluid sampling, electrical geophysics, and age determinations of hot-spring deposits. *J. Volcanol. Geotherm. Res.* **48**, 229–263 (1991).

21. G. Lucic, J. Stix, B. Wing, Structural controls on the emission of magmatic carbon dioxide gas, Long Valley Caldera, USA. *J. Geophys. Res. Solid Earth* **120**, 2262–2278 (2015).

22. A. J. Hotovec-Ellis, D. R. Shelly, D. P. Hill, A. M. Pitt, P. B. Dawson, B. A. Chouet, Deep fluid pathways beneath Mammoth Mountain, California, illuminated by migrating earthquake swarms. *Sci. Adv.* **4**, eaat5258 (2018).

23. D. P. Hill, E. Montgomery-Brown, D. R. Shelly, A. F. Flinders, S. Prejean, Post-1978 tumescence at Long Valley Caldera, California: A geophysical perspective. *J. Volcanol. Geotherm. Res.* **400**, 106900 (2020).

24. W. Hildreth, Fluid-driven uplift at Long Valley Caldera, California: Geologic perspectives. *J. Volcanol. Geotherm. Res.* **341**, 269–286 (2017).

25. W. C. Hammond, C. Kreemer, I. Zaliapin, G. Blewitt, Drought-triggered magmatic inflation, crustal strain, and seismicity near the long Valley Caldera, Central Walker Lane. *J. Geophys. Res. Solid Earth* **124**, 6072–6091 (2019).

26. D. R. Shelly, J. L. Hardebeck, W. L. Ellsworth, D. P. Hill, A new strategy for earthquake focal mechanisms using waveform-correlation-derived relative polarities and cluster analysis: Application to the 2014 Long Valley Caldera earthquake swarm. *J. Geophys. Res. Solid Earth* **121**, 8622–8641 (2016).

27. G. A. Gualda, M. S. Ghiorso, The Bishop Tuff giant magma body: An alternative to the Standard Model. *Contrib. Mineral. Petrol.* **166**, 755–775 (2013).

28. T. W. Sisson, C. R. Bacon, Gas-driven filter pressing in magmas. *Geology* **27**, 613–616 (1999).

29. P. Dawson, J. Evans, H. Iyer, Teleseismic tomography of the compressional wave velocity structure beneath the Long Valley Region, California. *J. Geophys. Res. Solid Earth* **95**, 11021–11050 (1990).

30. D. Seccia, C. Chiarabba, P. De Gori, I. Bianchi, D. Hill, Evidence for the contemporary magmatic system beneath Long Valley Caldera from local earthquake tomography and receiver function analysis. *J. Geophys. Res. Solid Earth* **116**, B12314 (2011).

31. G. Lin, Seismic velocity structure and earthquake relocation for the magmatic system beneath Long Valley Caldera, eastern California. *J. Volcanol. Geotherm. Res.* **296**, 19–30 (2015).

32. A. F. Flinders, D. R. Shelly, P. B. Dawson, D. P. Hill, B. Tripoli, Y. Shen, Seismic evidence for significant melt beneath the Long Valley Caldera, California, USA. *Geology* **46**, 799–802 (2018).

33. Z. Zhan, Distributed acoustic sensing turns fiber-optic cables into sensitive seismic antennas. *Seismol. Res. Lett.* **91**, 1–15 (2020).

34. N. J. Lindsey, E. R. Martin, Fiber-optic seismology. *Annu. Rev. Earth Planet. Sci.* **49**, 309–336 (2021).

35. P. Jousset, G. Currenti, B. Schwarz, A. Chalari, F. Tilmann, T. Reinsch, L. Zuccarello, E. Privitera, C. M. Krawczyk, Fibre optic distributed acoustic sensing of volcanic events. *Nat. Commun.* **13**, 1753 (2022).

36. O. G. Flovenz, R. Wang, G. P. Hersir, T. Dahm, S. Hainzl, M. Vassileva, V. Drouin, S. Heimann, M. P. Isken, E. Á. Gudnason, K. Ágústsson, T. Ágústsdóttir, J. Horálek, M. Motagh, T. R. Walter, E. Rivalta, P. Jousset, C. M. Krawczyk, C. Milkereit, Cyclical geothermal unrest as a precursor to Iceland's 2021 Fagradalsfjall eruption. *Nat. Geosci.* **15**, 397–404 (2022).

37. A. Fichtner, S. Klaasen, S. Thrastarson, Y. Çubuk-Sabuncu, P. Paitz, K. Jónsdóttir, Fiber-optic observation of volcanic tremor through floating ice sheet resonance. *Seismic Rec.* **2**, 148–155 (2022).

38. E. Biondi, X. Wang, E. F. Williams, Z. Zhan, Geolocalization of large-scale DAS channels using a GPS-tracked moving vehicle. *Seismol. Soc. Am.* **94**, 318–330 (2023).

39. F. Waldhauser, D. Schaff, Large-scale relocation of two decades of Northern California seismicity using cross-correlation and double-difference methods. *J. Geophys. Res.* **113**, B08311 (2008).

40. E.-J. Lee, P. Chen, T. H. Jordan, P. B. Maechling, M. A. M. Denolle, G. C. Beroza, Full-3-D tomography for crustal structure in Southern California based on the scattering-integral and the adjoint-wavefield methods. *J. Geophys. Res. Solid Earth* **119**, 6421–6451 (2014).

41. J. R. Peacock, M. T. Mangan, D. McPhee, P. E. Wannamaker, Three-dimensional electrical resistivity model of the hydrothermal system in Long Valley Caldera, California, from magnetotellurics. *Geophys. Res. Lett.* **43**, 7953–7962 (2016).

42. I. Koulakov, M. Bohm, G. Asch, B. G. Lühr, A. Manzanares, K. S. Brotopuspito, P. Fauzi, M. A. Purbawinata, N. T. Puspito, A. Ratdomopurbo, H. Kopp, W. Rabbel, E. Shevkunova, PandSvelocity structure of the crust and the upper mantle beneath central Java from local tomography inversion. *J. Geophys. Res. Solid Earth* **112**, 1–19 (2007).

43. S. Hurwitz, C. D. Farrar, C. F. Williams, The thermal regime in the resurgent dome of Long Valley Caldera, California: Inferences from precision temperature logs in deep wells. *J. Volcanol. Geotherm. Res.* **198**, 233–240 (2010).

44. K. Sieh, M. Bursik, Most recent eruption of the Mono Craters, eastern central California. *J. Geophys. Res. Solid Earth* **91**, 12539–12571 (1986).

45. L. C. Pakiser, F. Press, M. F. Kane, Geophysical investigation of Mono Basin, California. *Geol. Soc. Am. Bull.* **71**, 415–448 (1960).

46. J. R. Peacock, M. T. Mangan, D. McPhee, D. A. Ponce, Imaging the magmatic system of Mono Basin, California, with magnetotellurics in three dimensions. *J. Geophys. Res. Solid Earth* **120**, 7273–7289 (2015).

47. R. Maguire, B. Schmandt, M. Chen, C. Jiang, J. Li, J. Wilgus, Resolving continental magma reservoirs with 3D surface wave tomography. *Geochem. Geophys. Geosyst.* **23**, e2022GC010446 (2022).

48. D. P. Hill, Structure of Long Valley Caldera, California, from a seismic refraction experiment. *J. Geophys. Res.* **81**, 745–753 (1976).

49. A. Stroujkova, P. Malin, A magma mass beneath Casa Diablo? Further evidence from reflected seismic waves. *Bull. Seismol. Soc. Am.* **90**, 500–511 (2000).

50. N. Nakata, D. R. Shelly, Imaging a crustal low-velocity layer using reflected seismic waves from the 2014 earthquake swarm at Long Valley Caldera, California: The magmatic system roof?. *Geophys. Res. Lett.* **45**, 3481–3488 (2018).

51. E. Montgomery-Brown, C. W. Wicks Jr., P. F. Cervelli, J. O. Langbein, J. L. Svarc, D. R. Shelly, D. P. Hill, M. Lisowski, Renewed inflation of Long Valley Caldera, California (2011 to 2014). *Geophys. Res. Lett.* **42**, 5250–5257 (2015).

52. R. B. Herrmann, Computer programs in seismology: An evolving tool for instruction and research. *Seismol. Res. Lett.* **84**, 1081–1088 (2013).

53. T. M. Brocher, Empirical relations between elastic wavespeeds and density in the Earth's crust. *Bull. Seismol. Soc. Am.* **95**, 2081–2092 (2005).

54. C. Jiang, B. Schmandt, J. Farrell, F. C. Lin, K. M. Ward, Seismically anisotropic magma reservoirs underlying silicic calderas. *Geology*, **46**, 727–730 (2018).

55. W. Zhu, E. Biondi, J. Li, Z. E. Ross, Z. Zhan, Seismic arrival-time picking on distributed acoustic sensing data using semi-supervised learning. arXiv:2302.08747 [physics.geo-ph] (17 February 2023).

56. W. Zhu, G. C. Beroza, PhaseNet: A deep-neural-network-based seismic arrival-time picking method. *Geophys. J. Int.* **216**, 261–273 (2019).

57. O. Ronneberger, P. Fischer, T. Brox, *International Conference on Medical Image Computing and Computer-Assisted Intervention* (Springer, 2015), pp. 234–241.

58. J. C. VanDecar, R. S. Crosson, Determination of teleseismic relative phase arrival times using multi-channel cross-correlation and least squares. *Bull. Seismol. Soc. Am.* **80**, 150–169 (1990).

59. J. Li, W. Zhu, E. Biondi, Z. Zhan, Earthquake focal mechanisms with distributed acoustic sensing. *Nat. Commun.* **14**, 4181 (2023).

60. H. Zhang, C. Thurber, Development and applications of double-difference seismic tomography. *Pure Appl. Geophys.* **163**, 373–403 (2006).

61. R.-E. Plessix, A review of the adjoint-state method for computing the gradient of a functional with geophysical applications. *Geophys. J. Int.* **167**, 495–503 (2006).

62. S. Li, A. Vladimirsky, S. Fomel, First-break traveltime tomography with the double-square-root eikonal equation. *Geophysics* **78**, U89–U101 (2013).

63. P. Tong, Adjoint-state traveltime tomography: Eikonal equation-based methods and application to the Anza area in Southern California. *J. Geophys. Res.* **126**, e2021JB021818 (2021).

64. E. Biondi, G. Barnier, R. G. Clapp, F. Picetti, S. Farris, An object-oriented optimization framework for large-scale inverse problems. *Comput. Geosci.* **154**, 104790 (2021).

65. M. C. White, H. Fang, N. Nakata, Y. Ben-Zion, PyKonal: A python package for solving the eikonal equation in spherical and cartesian coordinates using the fast marching method. *Seismol. Res. Lett.* **91**, 2378–2389 (2020).

66. F. Waldhauser, W. L. Ellsworth, A double-difference earthquake location algorithm: Method and application to the northern Hayward Fault, California. *Bull. Seismol. Soc. Am.* **90**, 1353–1368 (2000).

67. D. C. Liu, J. Nocedal, On the limited memory BFGS method for large scale optimization. *Math. Program.* **45**, 503–528 (1989).

68. P. Small, D. Gill, P. J. Maechling, R. Taborda, S. Callaghan, T. H. Jordan, K. B. Olsen, G. P. Ely, C. Goulet, The SCEC unified community velocity model software framework. *Seismol. Res. Lett.* **88**, 1539–1552 (2017).

69. C. A. Zelt, Lateral velocity resolution from three-dimensional seismic refraction data. *Geophys. J. Int.* **135**, 1101–1112 (1998).

70. H. H. Huang, Y. M. Wu, X. Song, C. H. Chang, S. J. Lee, T. M. Chang, H. H. Hsieh, Joint Vp and Vs tomography of Taiwan: Implications for subduction-collision orogeny. *Earth Planet. Sci. Lett.* **392**, 177–191 (2014).
